# ARID3B increases ovarian tumor burden and is associated with a cancer stem cell gene signature

**DOI:** 10.18632/oncotarget.2247

**Published:** 2014-07-23

**Authors:** Lynn Roy, Serene J. Samyesudhas, Martin Carrasco, Jun Li, Stancy Joseph, Richard Dahl, Karen D. Cowden Dahl

**Affiliations:** ^1^ Department of Biochemistry and Molecular Biology, Indiana University School of Medicine, South Bend, Indiana; ^2^ Department of Applied and Computational Mathematics and Statistics, University of Notre Dame, Notre Dame, Indiana; ^3^ Department of Microbiology and Immunology, Indiana University School of Medicine, South Bend, Indiana; ^4^ Department of Chemistry and Biochemistry and Eck Institute for Global Health, Notre Dame University, Notre Dame, Indiana; ^5^ Indiana University Melvin and Bren Simon Cancer Center, Indianapolis, Indiana

**Keywords:** Ovarian cancer, transcription factor, cancer stem cells, metastasis, xenografts

## Abstract

Ovarian cancer is the most deadly gynecological malignancy since most patients have metastatic disease at the time of diagnosis. Therefore, identification of critical pathways that contribute to ovarian cancer progression is necessary to yield novel therapeutic targets. Recently we reported that the DNA binding protein ARID3B is overexpressed in human ovarian tumors. To determine if ARID3B has oncogenic functions *in vivo*, ovarian cancer cell lines stably expressing ARID3B were injected intraperitoneally into nude mice. Overexpression of ARID3B increased tumor burden and decreased survival. To assess how ARID3B contributes to the increased tumor growth *in vivo*, we identified ARID3B induced genes in tumor ascites cells. ARID3B induced expression of genes associated with metastasis and cancer stem cells (*CD44*, *LGR5*, *PROM1* (CD133), and *Notch2*). Moreover, ARID3B increased the number of CD133+ (a cancer stem cell marker) cells compared to control cells. The increase in CD133+ cells resulting from ARID3B expression was accompanied by enhanced paclitaxel resistance. Our data demonstrate that ARID3B boosts production of CD133+ cells and increases ovarian cancer progression *in vivo*.

## INTRODUCTION

Ovarian cancer is the leading cause of death from a gynecological malignancy and resulted in an estimated 14,030 deaths in 2013 (American Cancer Society Facts and Figures 2013). Patients initially respond well to chemotherapy, but most patients will relapse after the initial platinum/taxane therapy [1]. Understanding the molecular mechanisms that drive malignancy is critical for diagnosing and developing effective treatments for this devastating disease.

Data indicate that cancer stem cells (CSC) contribute to chemoresistance. CSCs are a subset of slow cycling and undifferentiated cells that divide asymmetrically to generate highly proliferative, invasive, and chemoresistant tumor cells [2]. Following chemotherapy, the CSCs survive and proliferate generating chemoresistant tumors. CSCs have been isolated by a number of groups from malignant ascites from ovarian cancer patients [3-11]. These cells express canonical stem cell markers, are chemoresistant, and tumorigenic. One of the most commonly reported CSC markers is CD133. CD133 expression is low in normal ovary and benign tumors compared to malignant ovarian cancer [12]. Furthermore CD133+ cells are more tumorigenic than CD133- cells, suggesting that the CD133 population has stem cell properties [13].

Recently we reported that the transcription factor ARID3B, a member of the AT-rich interactive (ARID) family [14], is overexpressed in serous ovarian cancer [15]. In addition to being overexpressed in ovarian cancer and late stage neuroblastoma [16], ARID3B is highly expressed in embryonic (ES) and induced pluripotent stem cells [17, 18]. In ES cells ARID3B interacts with the stem cell factors Oct4 and Nanog [18]. Therefore we hypothesized that ARID3B may contribute to ovarian cancer growth by impacting cancer stem cell development.

To address this hypothesis, we assessed the role of ARID3B in ovarian tumor growth and determined the effects of ARID3B overexpression on gene expression and drug resistance. We generated a xenograft mouse model of ovarian cancer that recapitulates the level of ARID3B overexpression found in human tumor sections. We found that ARID3B increases tumor growth *in vivo* and induces CSC genes in tumor ascites. Furthermore, ARID3B overexpression increases the pool of CD133+ spheroids in ovarian cancer cell lines. This study demonstrates for the first time that ARID3B promotes ovarian tumor development in part by regulating stem cell genes.

## RESULTS

### Generation of xenograft mouse model of ARID3B overexpression in SKOV3IP ovarian cancer cells

**Figure 1:**
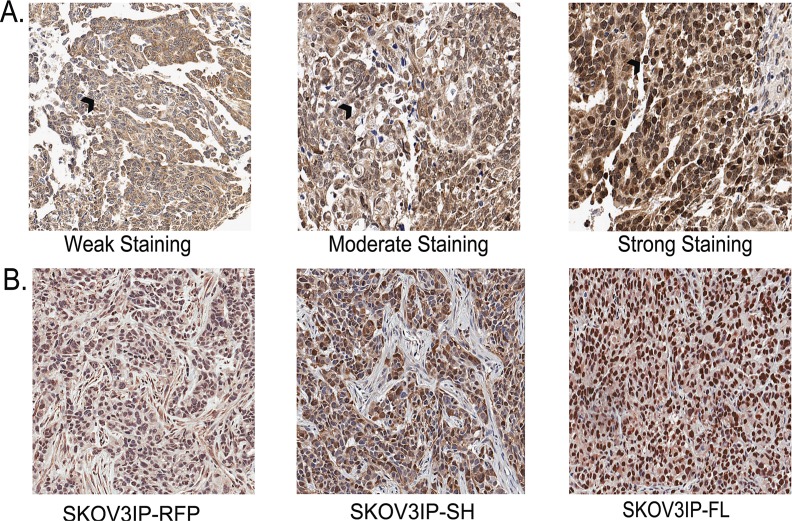
SKOV3IP xenograft tumors recapitulate the expression of ARID3B in ovarian cancer (A) Immunohistochemistry was performed on tissue microarrays containing 102 cases of ovarian cancer. TMAs were scored for ARID3B expression. Representative images of light staining for ARID3B staining: (clear cell carcinoma, grade 2, stage IIC), moderate (serous, grade 3, stage IIIC), or strong (serous, grade3, stage IV). Black arrow indicates nucleus. Original magnification is 20x. (B) IHC was performed on xenografts in nude mice from SKOV3IP-RFP, SKOV3-ARID3BSH, and SKOV3IP-ARID3BFL cells.

Recently we demonstrated that ARID3B is significantly overexpressed in ovarian cancer compared to normal ovarian surface epithelium and benign ovarian tumors [19]. We wanted to establish a xenograft model of ovarian cancer in mice where ARID3B is expressed at comparable levels to what is observed in human tumors. To do this we analyzed the expression levels of ARID3B in human ovarian cancer by performing immunohistochemistry (IHC) for ARID3B on ovarian cancer tissue microarrays (TMAs). The TMAs contained duplicate samples from 102 different patients of which 45 were serous. Unlike our previous TMA analysis that was performed on commercially available TMAs, this TMA data set had patient outcome with regards to tumor relapse, survival, and time until survival. Using Aperio software, we were able to blindly quantify the percentage of cells with low, moderate, and high nuclear or cytoplasmic staining. In order to rule out background staining, we only further analyzed moderate and high staining (Figure 1A). We found that out of 102 patients, 60 had moderate nuclear ARID3B and relapsed; this correlation was significant (p=0.025, t-test). Interestingly strong nuclear staining did not correlate with relapse (p=0.15). We also found that moderate nuclear ARID3B correlated significantly with decreased time until relapse (p=0.029). We found no correlation between ARID3B and stage, histological type, or survival.

To recapitulate the overexpression of nuclear ARID3B found in the human tumors we generated xenografts in nude mice with ARID3B overexpressing SKOV3IP cells. Refer to the nuclear expression of ARID3B in Fig. 1. The SKOV3IP cell line is derived from ascites cells that developed in a mouse injected intraperitoneally (IP) with SKOV3 cells [20]. In a pilot study, we transduced SKOV3IP cells with lentivirus containing red fluorescent protein (RFP) or each of the two ARID3B splice forms. ARID3B has an alternative splice form, ARID3BSH. Since it lacks 81% of the DNA binding domain [15] it was included in our analysis as a negative control. These cells are referred to as SKOV3IP-RFP, SKOV3IP-ARID3BFL, and SKOV3IP-ARID3BSH. Cells from each cohort were injected IP into nude mice and were allowed to grow for 3 weeks. By 3 weeks the SKOV3IP-ARID3BFL cells formed large tumors, mice were euthanized, and tumors were fixed. IHC was performed to compare the expression of ARID3B in SKOV3IP-ARID3BFL tumors to human tumors. This analysis showed that the level of nuclear ARID3B in SKOV3IP-ARID3BFL tumors was similar to what was observed in 88% of human ovarian tumors (Figure 1A and B). The SKOV3IP-ARID3B xenograft tumors mimic the overexpression of ARID3B found in human ovarian cancer.

### ARID3BFL increases tumor burden *in vivo*

**Figure 2:**
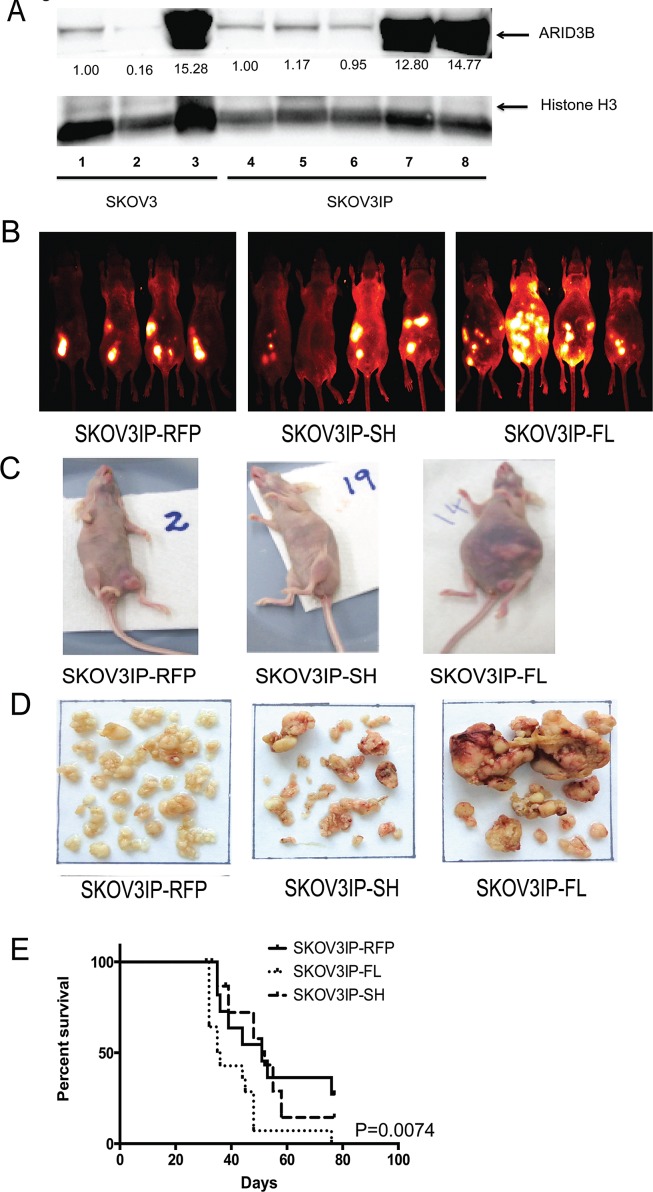
ARID3BFL accelerates tumor growth and decreases survival (A) Representative western blot was performed for ARID3BFL and Histone H3 in SKOV3 or SKOV3IP parental cells (lanes 1 and 4), RFP control cells (lanes 2, 5, and 6), and cells stably expressing ARID3BFL (lanes 3, 7 and 8). Densitometry (fold-change) is indicated under lanes. (B) Live *in vivo* fluorescent imaging of mice injected with SKOV3IP-RFP, SKOV3IP-ARID3BSH, or SKOV3IP-ARID3BFL cells. The live fluorescent images were obtained 31d post IP injection via the Kodak Multispectral FX. (C) Digital photographs of representative mice bearing xenograft tumors (SKOV3IP-RFP, SKOV3IP-ARID3BFL, and SKOV3IP-ARID3BSH) were taken. (D) Representative SKOV3IP-RFP, SKOV3IP-ARID3BFL, and SKOV3IP-ARID3BSH xenograft tumors. (E) Kaplan-Meier curve demonstrating that the median survival for SKOV3IP-ARID3BFL tumor bearing mice is significantly shorter (36 days) than SKOV3IP-RFP tumor (51 days), and SKOV3IP-ARID3BSH (52 days) (P=0.0074).

In our initial xenograft study we observed that ARID3BFL expressing tumors were considerably larger than the controls. We therefore repeated this study to determine how ARID3B influenced tumor growth, survival, and gene expression. We performed 2 separate experiments using cells that were transduced with RFP or ARID3B. Western blot of the SKOV3IP cells in both experiments is in shown in Figure 2A (compare parental and RFP controls in lanes 4-6 to SKOV3IP-ARID3BFL cells in lanes 7 and 8). Female nude (nu/nu) mice were IP injected with 10^6^ SKOV3IP-RFP, SKOV3IP-ARID3BFL, or SKOV3IP-ARID3BSH cells. Beginning 10 days after IP injection of the SKOV3IP tumor cells, fluorescent *in vivo* imaging of tumor growth was conducted weekly. A representative image of four mice from each of the three groups at 31 days post injection (Figure 2B) demonstrates that the mice injected with SKOV3IP-ARID3BFL cells developed large tumors earlier than the mice injected with SKOV3IP-RFP or SKOV3IP-ARID3BSH cells. A montage of images of representative mice between 18-39d (when all the SKOV3IP-ARID3BFL injected mice had died) is shown in Supplemental Figure 1. In contrast to the SKOV3IP-RFP and SKOV3IP-ARID3BSH injected mice, SKOV3IP-ARID3BFL mice also frequently presented with distended abdomens with large tumors visible beneath the skin (Figure 2C).

Mice were euthanized when they progressed to the point where the tumors were very large. Then the tumors were isolated and dissected from the organs. Figure 2D shows the representative tumor burden from each group of mice. Quantification of tumor weight revealed that the SKOV3IP-ARID3BFL tumors were on average more than 2.5x larger than those collected from the SKOV3IP-RFP mice. The average weights of the tumors were 1.91g+/-0.33 for SKOV3IP-RFP tumors, 3.22g+/-0.47 for SKOV3IP ARID3BSH tumors, and 5.46g+/-0.49 for SKOV3IP-ARID3BFL tumors (Supplemental Table 1). Survival analysis showed that the median survival for SKOV3IP-ARID3BFL tumor bearing mice was significantly reduced compared to mice bearing SKOV3IP-RFP or SKOV3IP-ARID3BSH tumors (RFP=51 days, ARID3BSH=52 days, ARID3BFL=36 days; P=0.0074) (Figure 2E). Collectively, these data demonstrate that overexpression of ARID3BFL results in a more aggressive ovarian cancer phenotype *in vivo*.

### ARID3B increases cell adhesion but not proliferation or invasion

We wanted to define the mechanisms by which ARID3B increases tumorigenesis. We performed IHC analysis for proliferation marker Ki67 and apoptosis marker cleaved Parp1 (Supplemental Figure 2) on RFP and ARID3BFL tumors. We did not find any difference in Ki67 staining. We actually detected more cleaved Parp1 in the ARID3B overexpressing tumors. We believe this was a due to the tumors being quite large and having large avascular regions. We counted the number of blood vessels in sections of SKOV3IP-RFP and SKOV3IP-ARID3BFL xenografts and found no statistical difference between the two. These data suggest that the increase in tumor burden resulting from ARID3B expression is not increased proliferation or decreased apoptosis.

We next assessed if ARID3B enhances tumor development *in vivo* by altering cells intrinsic phenotypes. We analyzed SKOV3 and SKOV3IP parental, RFP, ARID3BFL, and ARID3BSH expressing cells for changes in proliferation and invasion. We discovered that ARID3BFL and ARID3BSH did not affect proliferation (Supplemental Figure 3A, B) or invasion *in vitro* (Supplemental Figure 3C, D). We found that the cells expressing ARID3BFL had increased cellular projections and altered actin organization (Supplemental Figure 4A, B). Furthermore, ARID3BFL but not ARID3BSH increased adhesion to a wide range of extracellular matrix proteins (Supplemental Figure 4C) and increased vitronectin haptotaxis (Supplemental Figure 4D). The results indicate that ARID3B alters cell-matrix adhesion, which may contribute to increased tumor growth *in vivo*.

**Figure 3:**
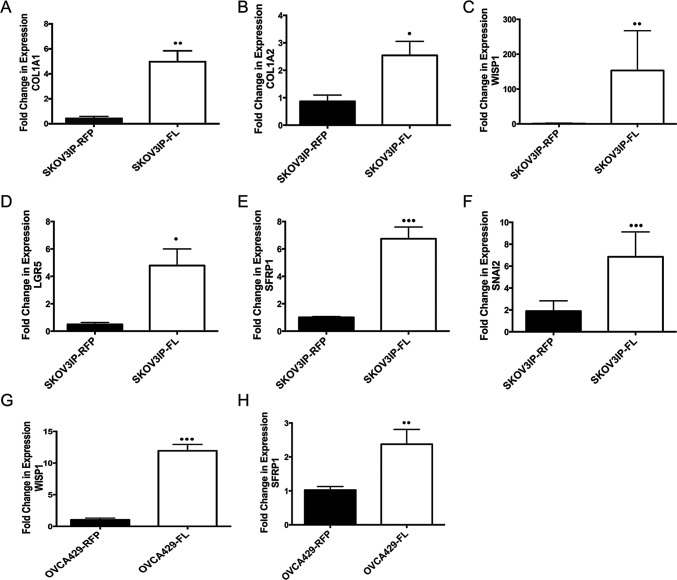
ARID3B induces collagen and cancer stem cell genes qRT-PCR was performed on ascites cells and peritoneal washes obtained from SKOV3IP-RFP (N=6) and SKOV3IP-ARID3BFL (N=13) mouse ascites. (A) COL1A1, (B) COL1A2, (C) WISP1, (D) LGFR5, (E) SFRP1, and (F) SNAI2 were all significantly increased in ascites derived from SKOV3IP-ARDI3BFL cells compared to SKOV3IP-RFP derived ascites. qRT-PCR was performed on OVCA429-RFP and OVCA429-ARDI3BFL cells for (G) WISP1 and (H) SFRP1. (* P ≤ 0.05) (** P ≤ 0.01) (*** P ≤ 0.001)

### ARID3B induces expression of cancer stem cell associated genes

ARID3B is a DNA binding protein [14, 21], however how ARID3B regulates tumor pathways has not been investigated. Therefore, we performed a microarray to identify putative ARID3B regulated genes that are induced by ARID3B in our tumor model. For this study RNA was isolated from ascites cells present in the mouse xenografts. We chose to use these cells because ascites fluid contains CSCs, which contribute to aggressive tumor behavior and because ARID3B is found in stem cell populations [3-11]. Isolated ascites cells were subsequently cultured *ex vivo* to remove non-tumor cells. We confirmed that the *ex vivo* cultures contained only SKOV3IP-RFP or SKOV3IP-ARID3BFL cells by examining the cells for RFP expression via fluorescent microscopy. Microarray was performed on RNA isolated from ascites cells from three independent SKOV3IP-RFP injected mice and three independent SKOV3IP-ARID3BFL injected mice. The top 35 upregulated genes in the SKOV3IP-ARID3BFL ascites are shown in Supplemental Table 2. Pathway analysis using Ingenuity Systems IPA software demonstrates that the top three biological functions that are associated with ARID3BFL induced genes are Cell Death and Survival, Cellular Movement, and Cancer. We used qRT-PCR to confirm that two of the most highly induced genes *COL1A1* and *COL1A2* were indeed induced in our ascites samples from SKOV3IP-ARID3BFL tumors compared to the RFP tumors (Figure 3A and B). Alterations in the expression of collagen genes are associated with metastasis in ovarian cancer [22]. Importantly *COL1A1* is both associated with CSCs and contributes to ovarian cancer chemoresistance [23, 24]. *COL1A2* exhibits altered expression in several tumor types most notably gastric cancer [25]. Interestingly, 6 of the top 35 genes have been implicated in CSCs (supplemental table 2). Table 1 lists ARID3B induced CSC genes including *ST3GAL6, ADAM19, and HTATIP2*, which were shown to be part of the CSC signature found in serous ovarian cancer ascites [10]. To validate these findings qRT-PCR was performed for *WISP1, LGR5, SFRP1*, and *SNAI2*, which are in the Wnt signaling pathway that is implicated in CSCs [26, 27] and metastasis [28]. Expression of these genes was significantly increased in the ARID3BFL expressing tumor cells (Figure 3C-F). To determine whether ARID3BFL overexpression regulates these genes in other ovarian cancer cell lines, we performed qRT-PCR for *SFRP1* and *WISP1* on OVCA429 cells transduced with RFP or ARID3B. Expression of ARID3B in OVCA429 cells is shown in Supplemental Figure 5. As anticipated, ARID3B overexpressing OVCA429 cells exhibited significantly increased *SFRP1* and *WISP1* expression (Figure 3G and H). These data suggest that ARID3BFL regulates genes involved in CSC gene signature.

**Table 1:** Cancer stem cell genes induced by ARID3B in xenograft tumor ascites

Gene Symbol	Fold Change (microarray)
COLA1A	87.13
WISP1	46.6
PROM2	18.75
FN1	16.51
CA9	8.92
SFRP1	8.61
SOX9	7.91
SNAI2	6.66
ADAM19	6.21
ST3GAL6	4.35
CD44	3.84
LIF	3.19
HTATIP2	2.48
SMAD3	2.3
NOTCH2	1.85

### ARID3B increases the expression of cancer stem cell markers

**Figure 4:**
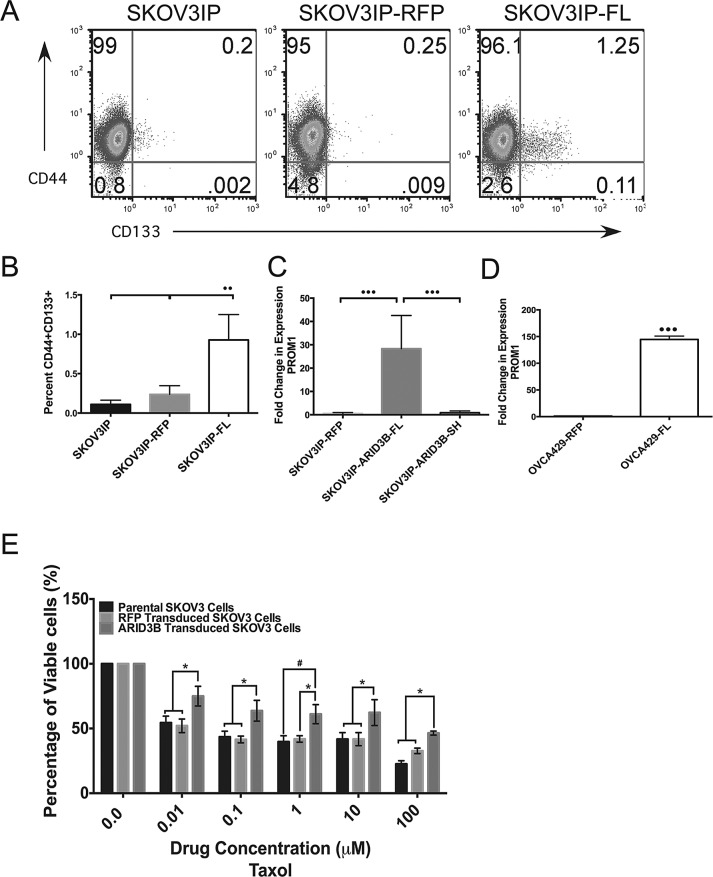
ARID3BFL expands the pool of ovarian cancer stem cells (A) Flow cytometry analysis of cell surface expression of CD44 and CD133 demonstrates an increase in CD44+/CD133+ cells in the SKOV3IP-ARID3BFL (N=5) cells compared to SKOV3IP (N=3) or SKOV3IP-RFP (N=3) cell populations. (SKOV3IP vs. SKOV3IP-ARID3BFL P=0.0037; SKOV3IP-RFP vs. SKOV3IP-ARID3BFL P=0.0097). Percentage of cells in each quadrant is indicated on scatter plots. (B) Graph indicating the increase in the percentage of CD44+/CD133+ cells found in SKOV3IP-ARID3BFL cells. SKOV3IP-ARID3BFL cells had an 82% and 69% increase in CD44+CD133+ over SKOV3IP and SKOV3IP-RFP cells respectively. (C) qRT-PCR performed on SKOV3IP-RFP, SKOV3IP-ARID3BFL, and SKOV3IP-ARID3BSH ascites cells show that PROM1 is significantly increased in SKOV3IP-ARID3BFL (N=11) cells with a 27-fold increase over both SKOV3IP-RFP (N=5) and SKOV3IP-ARID3BSH (N=12) cells. (D) qRT-PCR was performed on OVCA429-RFP and OVCA429-ARDI3BFL cells for PROM1. (E) SKOV3, SKOV3-RFP, and SKOV3-ARID3BFL cells were treated with increasing concentrations of paclitaxel. MTT assays were performed to measure viability after 48h. The data were analyzed using a Two-way ANOVA. (* P ≤ 0.05) (** P ≤ 0.01) (*** P ≤ 0.001)

The finding that ARID3B regulates stem cell associated genes is particularly intriguing as ARID3B is expressed in ES cells in a protein complex with the stem cell transcription factors Nanog, Oct4 and Nac1[18]. Since ARID3B is expressed in stem cells and associates with stem cell factors, we hypothesized that ARID3B promotes tumor growth through the regulation of a CSC phenotype. To determine if ARID3BFL increases production of CSCs, the cells that were injected into the nude mice to generate xenografts and the parental cells (SKOV3IP, SKOV3IP-RFP and SKOV3IP-ARID3BFL) cells were examined for the expression of the stem cell markers CD44 and CD133 using flow cytometry [29, 30]. Flow cytometry scatter plots in Figure 4A demonstrate the percentage of CD44+ (Y-axis) and CD133+ cells (X-axis). This analysis revealed that the cell populations expressing both CD44 and CD133 (PROM1) are significantly increased in the SKOV3IP-ARID3BFL cells (Figure 4A and B), suggesting that ARID3B increases the population of cells that express CD133 and therefore may be CSCs. In addition, qRT-PCR for *PROM1* demonstrated increased expression (p<0.001) in the cells from SKOV3IP-ARID3BFL ascites cells compared to SKOV3IP-RFP and SKOV3IP-ARID3BSH ascites cells (Figure 4C). Furthermore, ARID3BFL also increased *PROM1* gene expression in OVCA429 cells (Figure 4D). Thus these data suggest that ARID3B increases the population of CD133+ cells, that have been previously demonstrated to have enhanced tumorigenic capabilities[13].

Next, because CD133+ cells are associated with chemoresistance we ascertained the impact of ARID3B on drug resistance [7, 30, 31]. We chose to evaluate the effects of ARID3BFL overexpression on sensitivity to paclitaxel, which is used in first-line chemotherapy for ovarian cancer and SKOV3 cells already display resistance to cisplatin (the other drug used in first-line chemotherapy). ARID3BFL overexpressing cells showed a significant increase in viability compared to parental and RFP control cells when treated with 0.01-100µM paclitaxel for 48h (Figure 4E). We also evaluated cell for response to cisplatin and found no difference between parental, RFP, and ARID3B expressing cells (Supplemental Figure 6). Therefore, ARID3B overexpression enhances drug resistance, which is a well-known property of CSCs.

**Figure 5:**
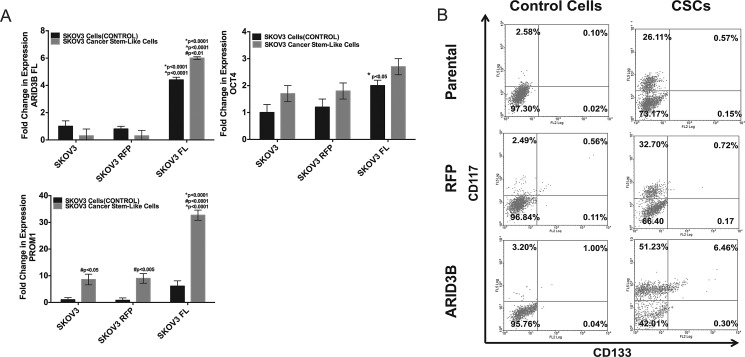
ARID3B enhances cancer stem cell selection in sphere-cultures (A) SKOV3, SKOV3-RFP, and SKOV3-ARID3BFL cells were enriched for CSCs by selection in cisplatin. qRT-PCR was performed for ARID3B, OCT4, and Prom1 on SKOV3, SKOV3-RFP, and SKOV3-ARID3BFL and CSCs isolated from each respective cell line. (B) Flow cytometry was performed for the stem cell markers CD117 and CD133 on Control Cells (not selected with cisplatin for CSCs) and CSCs (SKOV3 Parental), SKOV3-RFP (RFP), and SKOV3-ARID3BFL (ARID3B)). The percentage of CD117+ cells (Y-axis) and CD133+ cells (X-axis) is indicated for each quadrant. (* P ≤ 0.05) (** P ≤ 0.01) (*** P ≤ 0.001)

Lastly we assessed if ARID3B overexpression facilitates expansion of spheroids expressing stem cell markers using our modification of a published protocol [32], which is in press at Journal of Visualized Experiments (Cole, et al). We selected for chemoresistant ovarian cancer spheroids by culturing cells in cisplatin and subsequently culturing the non-adherent cells in a stem cell media. Viable spheroids expressed elevated CD133 (Figure 5). Exogenous ARID3B expression significantly increased the expression of *PROM1* in CSCs compared to non-CSCs as well as parental and RFP control CSCs (Figure 5A). ARID3B expression was associated with an increase in the percentage of CD117+/CD133+ CSCs in SKOV3 CSC cultures (Figure 5B). These studies indicate that ARID3B may contribute to ovarian cancer tumorigenesis and therapeutic resistance by regulating CSC production or function.

## DISCUSSION

We previously demonstrated that ARID3B is overexpressed in human serous ovarian cancer [19]. However, it was not known if ARID3B expression might enhance ovarian cancer tumorgenicity as it does for neuroblastoma [16]. To address the role of ARID3B in ovarian tumorigenesis, we performed mouse xenograft assays using SKOV3IP cells expressing exogenous ARID3BFL at levels similar to what is observed with IHC in human cancer. Our data demonstrates for the first time that ARID3B increases ovarian cancer tumor growth in a xenograft model of ovarian cancer. Mice injected with SKOV3IP-ARID3BFL cells have significantly more tumor burden and decreased survival compared to those injected with SKOV3IP-RFP or SKOV3IP-ARID3BSH cells. Although, the xenografts were only performed using SKOV3IP cells, we have found that ARID3B regulates CSC genes in OVCA429 cells. Additionally, we found that A2780 cells that expressed ARID3BFL formed subcutaneous tumors (4/5 mice) more efficiently than ones expressing RFP alone (1/5 mice). Future studies of ARID3B regulation of tumorigenesis and gene regulation in HGSOC cell lines are ongoing and will enable us to better dissect the contribution of ARID3B to ovarian cancer.

Many of the mice bearing SKOV3IP-ARID3BFL xenografts developed distended abdomens filled with ascites. Because ascites are a rich source of CSCs and ARID3B had previously been implicated in stem cell function[18], we explored the possibility that ARID3B increases CSC production. Microarray analysis on ascites cells from SKOV3IP-RFP or SKOV3IP-ARID3BFL xenografts identified that ARID3B overexpression is associated with elevated expression of many stem cell markers and metastasis associated genes. We observed up-regulation of genes in the Wnt signaling pathway (*LGR5, SFRP1*, and *WISP1*), which is associated with CSCs [27, 30, 33]. Similarly ARID3B overexpressing cells exhibited increased expression of genes (*ST3GAL6, ADAM19*, and *HTATIP2*) reported to be part of the serous ovarian cancer ascites CSC signature [10]. The increase in CSCs in culture corresponded to an increase in paclitaxel resistance. This is particularly interesting since CD133 is significantly elevated in recurrent platinum-resistant tumors [30]. Our results suggest that overexpression of ARID3BFL increases the percentage of tumor cells that posses “stem like” qualities and this leads to resistance to paclitaxel therapy.

Since ARID3B is a DNA binding protein, we predict that some of the phenotypes that are attributed to ARID3B expression and activation are through its ability to directly and indirectly regulate gene transcription. Ongoing studies will determine which stem cell genes are directly regulated by ARID3B promoter association. Interestingly, with ARID3B overexpression we see a substantial increase in CD133 mRNA; however, while we only observe a small yet significant increase in CD133+ (cell surface) cells (from 0.2-1.25%). More cells are expressing CD133 mRNA than have the “stem-cell phenotype”. This suggests that the CD133 mRNA may not be translated or transported to the cell surface, the reason for this is not known. The role of CD133 in stem cell production or function is not clear and warrants future investigations. Alternatively, ARID3B may activate a stem cell program, but progeny cells further divide and “differentiate” or lose their stem cell markers. This may impart explain the heterogeneity in the tumor population. Therefore ARID3B induces stem cell genes, which may contribute to the increased tumor production in xenografts expressing ARID3BFL.

We found that moderate, but not high expression of ARID3B in the nucleus significantly correlates with ovarian cancer relapse. This agrees with our published data demonstrating that exceedingly high levels of ARID3B lead to TNF-mediated apoptosis [15]. In contrast, more moderate levels of overexpression (observed in the cell lines used in these studies) leads to a more aggressive form of ovarian cancer. In conclusion, our studies reveal that ARID3BFL increases the aggressiveness of ovarian tumor formation and decreases survival time *in vivo*. We propose that one mechanism for ARID3B enhancement of tumor growth is through activation of genes involved in CSC pathways leading to CSC production and paclitaxel resistance.

## Materials & Methods

### Immunohistochemistry (IHC) and H&E

Three TMAs containing ovarian cancer sections were provided by Dr. Tanja Pejovic (Oregon Health and Science University). These TMAs contained section from102 different cases. IHC was performed on paraffin embedded tumors according to the staining procedure provided by the VECTASTAIN ABC KIT using ImmPACT DAB and Hematoxylin QS as a counterstain (Vector Laboratories, Burlingame, CA). Anti-ARID3B IHC Antibody (Bethyl Laboratories, Montgomery, TX), anti-KI67 (Novus Biologicals, Littleton, CO), and Cleaved PARP (Asp214) (Cell Signaling Technology, Danvers, MA) were used. Slides were scanned at the 20x magnification setting using Aperio Scan Scope (Vista, CA). Hematoxylin and eosin (H&E) staining was performed on xenograft tumors from SKOV3IP-RFP and SKOV3IP-RFP cells injections according to standard procedures.

### Cell Culture

SKOV3 cells were obtained from ATCC (Manassas, VA, USA) (8/2011) and maintained in McCoy's 5A medium with 0.1mM L-glutamine, 10% fetal bovine serum (FBS), 50U/ml penicillin and 50μg/ml streptomycin. SKOV3IP cell line (from Dr. Mills, MD Anderson Cancer Center), was maintained in RPMI-1640 medium supplemented with 0.1mM L-glutamine, 5% FBS, 50U/ml penicillin and 50μg/ml streptomycin, 1% non-essential amino acids, and 1mM Sodium pyruvate and Hygromycin B OVCA429 cells were provided by Dr. Bast (MD Anderson Cancer Center) and grown in minimal essential media with 0.1mM L-glutamine, 10% FBS, 50U/ml penicillin and 50μg/ml streptomycin. Red fluorescent protein (RFP), ARID3BFL and ARID3BSH were overexpressed using a lentiviral approach as described [15]. Antibiotic selection was not used as nearly 100% of the cells were transduced with the lentivirus. For *in vivo* and *in vitro* studies, cells were transduced with virus, expanded and injected into mice or used for other experiments within 2-3 weeks. Tissue culture reagents were obtained from Life Technologies, Carlsbad, CA. All cell lines were authenticated on October 1, 2013, ATCC by STR profiling.

### Western Blot Analysis

Whole cell lysates were prepared using RIPA buffer (50 mM Tris pH 7.5, 150 mM NaCl, 1% NP-40, 0.5% EDTA, 0.1% SDS and protease inhibitors). Bicinchoninic Acid (BCA) protein assay (Pierce, Rockford, IL) determined protein concentrations. Thirty µg of whole cell lysates were separated by SDS-PAGE and transferred to nitrocellulose membrane. Membranes were blotted with ARID3B antibody (Bethyl Laboratories, Montgomery, TX: Cat# A302-564A), and Histone H3 (D1H2) antibody (Cell Signaling, Danvers, MA) for loading control. Densitometry analysis was performed on three blots using BIORAD Chemidoc XRS+ System Image Lab Software (Hercules, CA).

### Xenograft mouse models of ovarian cancer

Under an approved IACUC protocol (ND #14-060), six-week-old female nude mice nu/nu (Charles River, Wilmington, MA) were maintained at the Freimann Life Science Center (University of Notre Dame). In the pilot study (4 mice per group), 2×10^6^ SKOV3IP-RFP, SKOV3IP-ARID3BFL, and SKOV3IP-ARID3BSH cells in 300 μl of phosphate buffered saline (PBS; 137 mM NaCl, 2.7 mM KCl, and 11.9 mM phosphate buffer, pH 7.4) were injected intraperitoneally (IP) into nude mice. Mice had visible tumors after 3 weeks and were euthanized. For the survival studies and analysis of tumor growth, 1×10^6^ SKOV3IP-RFP (9 mice), SKOV3IP-ARID3BFL (16 mice), and SKOV3IP-ARID3BSH (14 mice) cells in 300 μl of PBS cells were each injected into nude mice. Two separate experiments were conducted on independent transductions of the cells. Mice were imaged weekly. When the mice showed signs of illness they were imaged twice weekly and then euthanized. Ascites fluid was collected using a 25 G needle and 5 mL syringe. If no ascites fluid was present a peritoneal wash was performed by injecting 2 mL of PBS IP and then collecting the fluid. Fluorescent imaging of tumor growth in live animals were acquired at the Notre Dame Integrated Imaging Facility using the Multispectral FX (Carestream, Rochester, NY

### RNA extraction and qRT-PCR

Gene array (see Supplemental information) and qRT-PCR analysis was conducted on tumor derived ascites cells from SKOV3IP-RFP and SKOV3IP-ARID3BFL injected mice. Ascites cells were collected from the mice as described above. Half of the fluid was centrifuged to pellet the cells. The supernatant was removed and the cells were placed in 0.5 mL of TRIzol (Invitrogen, Carlsbad, CA). The remaining ascites fluid was cultured as described for SKOV3IP cells. The RNA was extracted using TRIzol according to the manufacturer's recommendations. cDNA was prepared using the High Capacity cDNA kit (Life Technologies, Carlsbad, CA). qRT-PCR was performed using Taqman master mix solution (Life Technologies, Carlsbad, CA) and primers for Col1A1, Col1A2, ARID3B, PROM1, LGR5, SFRP1, WISP1, and SNAI2 from Integrated DNA Technologies (Coralville, IA) and GAPDH from (Qiagen, Valencia, CA). qRT-PCR was conducted in duplicate using the Biorad CFX96 C1000 System (Biorad, Hercules, CA). Statistics were calculated using a one-way ANOVA.

### Flow Cytometry

To examine stem cell markers in SKOV3IP cells expressing ARID3BFL or control cells, flow cytometry was performed to detect CD133 and CD44 and data were analyzed with FlowJo software (Ashland, OR). SKOV3IP, SKOV3IP-RFP, and SKOV3IP-ARID3BFL cells were incubated with CD133/(AC133)-PE (Miltenyi Biotec, Auburn, CA) and anti-human CD44-APC (Biolegend, San Diego, CA). N=3. Statistics were calculated using a one-way ANOVA. Expression of CD117and CD133 was evaluated in SKOV3 CSC and SKOV3 (Control) cells that were either non-transduced or stably expressing RFP or ARID3B. Cells were harvested with Accumax (Millipore, Billerica, MA), and washed twice with cold PBS. Cells were incubated in their respective media for 1 h prior to labeling. Cells were suspended in 0.1%BSA/PBS and labeled with anti-CD 133-PE (Mitenyl Biotec, Auburn, CA) and anti-CD117-FITC (anti-CD117-Biotin; Cell Signaling, Danvers, MA and Streptavidin-FITC; Mitenyl Biotec, Auburn, CA) per the manufacturer's instructions. Cells were fixed overnight in 1% paraformaldehyde. Flow cytometry was performed using a Beckman Coulter FC500 Flow Cytometer (Brea, CA). N=3 Statistics were calculated using Two-Way Anova test.

### Enrichment for Cancer Stem Cells

CSC cultures using SKOV3, SKOV3-RFP, and SKOV3-ARID3B cells, were generated by treating cells with 20μM of cisplatin (Sigma, St. Louis, MO) for 72h. Surviving cells were trypsinized and cultured in serum free DMEM/F12 media (Life Technologies, Grand Island, NY) supplemented 5μg/ml insulin (Invitrogen, Carlsbad, USA), 10 ng/ml human recombinant epidermal growth factor (EGF; Invitrogen, Carlsbad, USA), 10 ng/ml basic fibroblast growth factor (bFGF, Invitrogen, Carlsbad, USA), 12 ng/ml leukemia inhibitory factor (LIF; Santa Cruz Biotechnologies, Santa Cruz, CA) and 0.4% bovine serum albumin (BSA; Sigma, St. Louis, MO). The selected cells formed non-adherent spheres when grown in this condition. The media was changed every 2 days. SKOV3 CSCs were collected for RNA analysis and flow cytometry.

### MTT Assay to measure response to paclitaxel treatment

Ovarian cancer cells were plated onto 96-well plates (5000 cell per well) overnight. Cells were treated with increasing concentrations of paclitaxel for 48h. Viable cells were assayed using the MTT (3-(4, 5-Dimethylthiazol-2-yl)-2, 5-diphenyltetrazolium bromide) assay according to the manufacturer's instructions (Sigma, St. Louis, MO). The plates were read at 595 nm, and the data were analyzed using SoftmaxPro Version 3.1.1 (Molecular Devices, Sunnyvale, CA). Experiments were performed in triplicate and statistics were calculated using a Two-Way Anova test.

### Statistics

All statistical analyses were performed using the Prism software (GraphPad Software, Inc., La Jolla, CA). The data are presented as the means ± SEM. Comparisons between experimental conditions and controls were made by one-way ANOVA or Two-Way ANOVA with and without repeated measures with either Tukey or Sidak posttests. Student t –tests were used in the comparison of ARID3B nuclear expression and tumor relapse. Animal studies were analyzed using the Kaplan-Meier survival analysis with log-rank significance test and correlation analysis. Statistical differences are denoted as follows: * P ≤ 0.05, ** P ≤ 0.01, *** P ≤ 0.001.

## SUPPLEMENTARY MATERIAL, FIGURES AND TABLES


