# Supplementary material for: ARID3B increases ovarian tumor burden and is associated with a cancer stem cell gene signature
Source: Oncotarget. 2014 Jul 23;5(18):8355–66. doi: 10.18632/oncotarget.2247 (PMC4226688; doi:10.18632/oncotarget.2247)
Supplement: Supplementary file 1 [file oncotarget-05-8355-s001.pdf]

ARID3B increases ovarian tumor burden and is associated with a cancer stem cell gene signature

Supplementary Material

Supplementary Table 1: Average Tumor Weight and Percent with Ascites Fluid by Cell Type

Cell Type	N	Average Tumor Weight (g)	Tumor weight P-values		% with Ascites Fluid	Ascites Fluid P-values
SKOV3IP-RFP	8	1.91	SKOV3IP-RFP vs SKOV3IP-SH	n.s.	37.5	n.s.
SKOV3IP-ARID3BFL	14	5.46	SKOV3IP-RFP vs SKOV3IP-FL	< 0.0001	42.92	
SKOV3IP-ARID3BSH	13	3.32	SKOV3IP-SH vs SKOV3IP-FL	0.0048	23.07	

Supplementary Table 2: Top 35 Genes Upregulated in SKOV3IP-ARID3BFL Ascites Cells

Top 35 Upregulated Genes		Fold Change
COL6A3	collagen, type VI, alpha 3	1277.96
COL1A1	collagen, type I, alpha 1	87.13
AGR2	anterior gradient 2 homolog (Xenopus laevis)	51.69
WISP1	WNT1 inducible signaling pathway protein 1	46.60
COL8A1	collagen, type VIII, alpha 1	38.41
SLC8A1	solute carrier family 8 (sodium/calcium exchanger), member 1	30.24
SLC14A1	solute carrier family 14 (urea transporter), member 1 (Kidd blood group)	29.83
COL1A2	collagen, type I, alpha 2	27.24
SORBS1	sorbin and SH3 domain containing 1	26.85
EGR3	early growth response 3	24.65
PROM2	prominin 2	18.75
LGR5	leucine-rich repeat containing G protein-coupled receptor 5	16.62
FN1	fibronectin 1	16.51
MGC4294	uncharacterized MGC4294	15.83
LRRC17	leucine rich repeat containing 17	14.43
AIM1	absent in melanoma 1	13.82
LOC100271840	uncharacterized LOC100271840	12.65
DOCK4	dedicator of cytokinesis 4	12.31
EMP1	epithelial membrane protein 1	12.22
NLGN1	neuroligin 1	11.76
RGCC	regulator of cell cycle	11.22
ANK1	ankyrin 1, erythrocytic	10.78
FRMD3	FERM domain containing 3	10.40
FLJ20518	FSHD region gene 2 family, member C pseudogene	9.76
LOC100130894	uncharacterized LOC100130894	9.59
MLF1IP	MLF1 interacting protein	9.41
CA9	carbonic anhydrase IX	8.92
CPM	carboxypeptidase M	8.76
ADAMTSL3	ADAMTS-like 3	8.62
SFRP1	secreted frizzled-related protein 1	8.61
FRMD4A	FERM domain containing 4A	8.55

AFAP1L2	actin filament associated protein 1-like 2	8.45
DOCK10	dedicator of cytokinesis 10	8.37
HNMT	Histamine N-Methyltransferase	8.33
GPR68	G Protein-Coupled Receptor 68	8.08

Fluorescence Microscopy and Phalloidin Staining

Live fluorescence images were collected on RFP expressing cells using the EVOS fluorescence microscope (Advanced Microscopy Group, Bothell, WA). Phalloidin staining was performed using Alexa Fluor® 488 Phalloidin according to manufacturer's instructions (Life Technologies, Grand Island, NY). Images were collected using the EVOS fluorescence microscope.

Cell Adhesion Assay

ECM Cell Adhesion Array Kit (Colorimetric) from Chemicon International (Temecula, CA) was used to assess adhesion according to instructions. Experiments were performed thrice in triplicate and statistics were calculated using a two way ANOVA with repeated measures and a Tukey's multiple comparisons test.

Vitronectin haptotaxis cell migration assay

QCM Haptotaxis Cell Migration Assay – Vitronectin (Colorimetric) was performed according to manufacturer's instructions (Millipore, Billerica, MA). Experiments were performed thrice in triplicate and statistics were calculated using a two way ANOVA with repeated measures and a Sidak multiple comparisons test.

Alamar Blue Cell Viability and Cell Proliferation Assay

To determine the cell viability, $1.5x10^5$ cells / ml were plated in a sterile 96 well tissue culture plate and incubated for 24 hrs at 37°C in an incubator supplied with 5% CO_2. Alamar Blue assay was performed according to manufacturer's instructions. In brief, Alamar Blue reagent (Life Technologies, city state) was added to samples and incubated at 37°C for 1-4 hours. The absorbance of Alamar blue reagent was read on a spectrophotometer at 570 nm using 600nm as the reference wavelength. Finally, results were analyzed by plotting absorbance readings versus cell type.

Matrigel Invasion assay

Assays were performed according to provided instructions using BD Biocoat Tumor Invasion System composed of BD Falcon FluoroBlok 24-Multiwell Insert Plate (8 micron pore size) coated with BD Matrigel Matrix, a BD Falcon 24 –well plate and lid (BD Bioscience, San Jose, CA). Cells were grown to ~80% confluence. Fluorescent pre-labeling of cells was done using 10µg/ml of BD $DilC_{12}(3)$ Fluorescent Dye in DMEM containing 10% FBS for 1 hour at 37°C. After pre-labeling, cell monolayers were prepared in serum-free DMEM at 1×10^5 cells/ml. Then 5×10^4 cells were added to the apical chambers. The plate was incubated for 24 hours at 37°C, 5% CO_2. Readings were taken at wavelengths of 549/565 nm (Ex/Em). Results were analyzed by plotting Fluorescence readings versus cell type.

Microarray

Three RNA samples each from SKOV3IP-RFP and SKOV3IP-ARID3BFL ascites fluid/peritoneal washes were prepared for microarray by using a RNA cleanup protocol (RNeasy Kit from Qiagen, Valencia, CA). The microarray was performed at the Notre Dame Genomics Core Facility on an Affymetrix Human Genome U133 Plus 2 GeneChip (Affymetrix, Santa Clara, CA). The probe set expression were normalized and summarized by using the GC robust multi-array average (GCRMA) algorithm(20). Control probe sets and probe sets that were "not detectable" were filtered out for further analysis. "Not detectable" was defined as probe sets that

are either being called “absent” by Affymetrix's Call Detection Algorithm (Manual titled “GeneChip Expression Analysis Data Analysis Fundamentals”, Affymetrix; www.affymetrix.com) in all of the six arrays, or having all its expression reported by GCRMA less than 2.5. Totally, 26274 out of 54675 of probe sets survived the filtering. Significance Analysis of Microarrays (SAM,(21)) was used for detecting the differentially expressed genes among the two groups of samples: RFP and FL. 1012 probe sets were found to be significant with false discovery rate (FDR) less than 10%. Of these probe sets, 199 were down regulated in ARID3BFL.

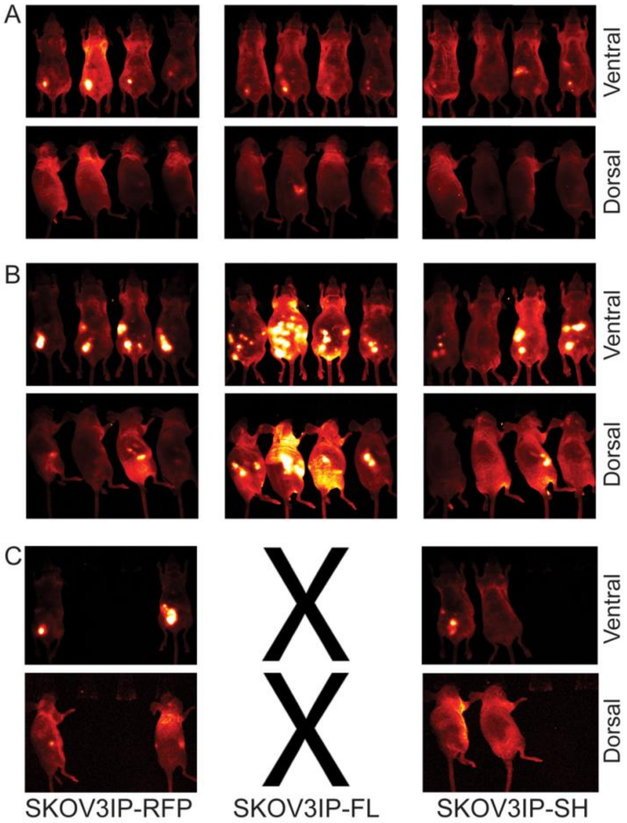

Supplemental Figure 1: Time course of in vivo fluorescent imaging. Ventral and dorsal view of representative mice from each group. (A) Tumor development at 18 d post injection. (B) SKOV3IP-ARID3BFL cells induced numerous tumors faster than either SKOV3IP-RFP or SKOV3IP-ARID3BSH cells. Image taken at 31 d post injection. (C) By 39 d post injection, all mice in this representative group injected with SKOV3IP-ARID3BFL cells had died. Two SKOV3IP-RFP and SKOV3IP-ARID3BSH injected mice had died.

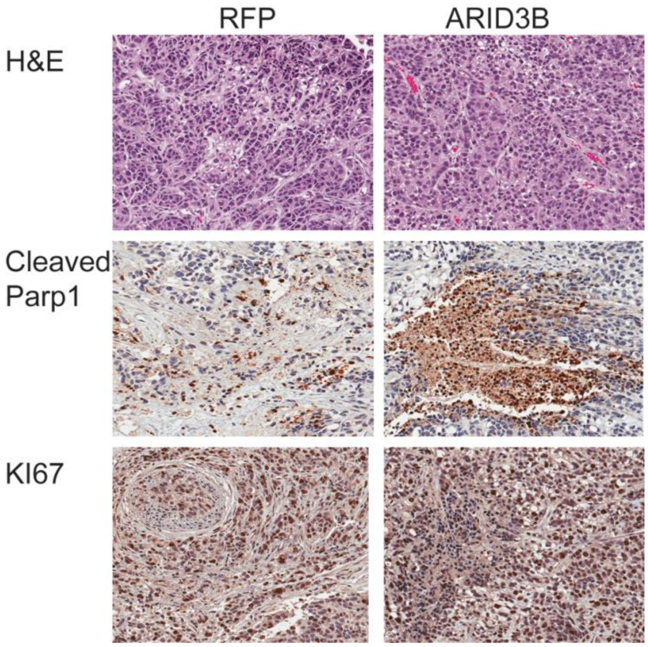

Supplemental Figure 2: Histology and IHC on xenograft tumors. Tumors from mice described in Fig.1 (SKOV3IP-RFP and SKOV3IP-ARID3BFL derived xenografts) were harvested and stained for H&E and IHC was performed for cleaved Parp1 (apoptosis) or KI67 (proliferation).

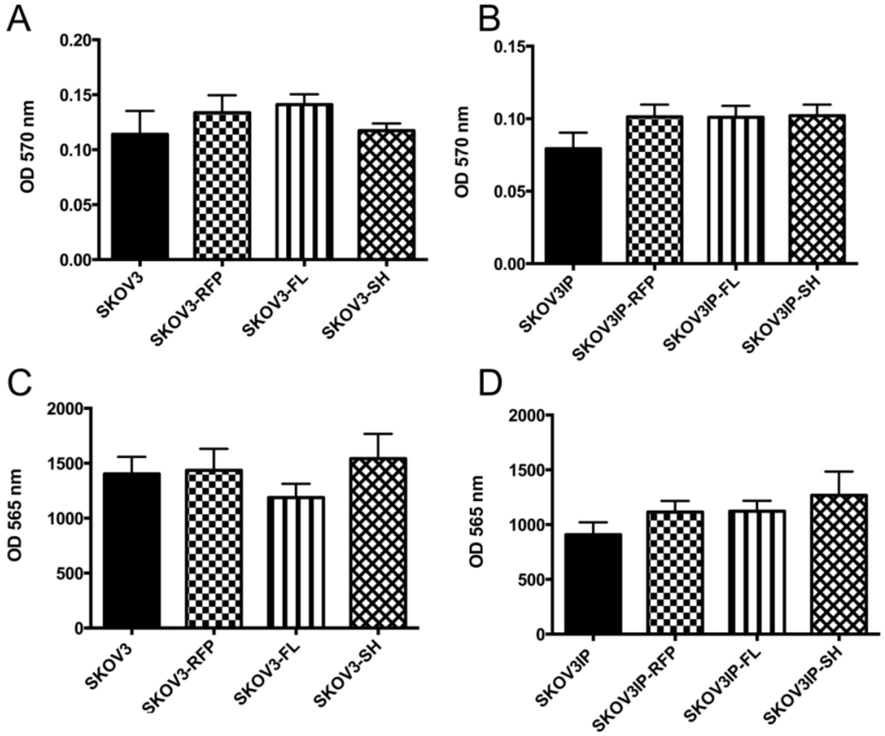

Supplemental Figure 3: ARID3B does not alter ovarian cancer cell proliferation or invasion. Alamar Blue cell viability assay was performed on SKOV3 (A) and SKOV3IP (B) cells alone or transduced with RFP, ARID3BFL, or ARID3BSH. There were no differences in proliferation between treatments. ARID3B expression does not alter SKOV3 (C) or SKOV3IP (D) cell invasion on cells on parental cells or cells transduced with RFP, ARID3BFL, or ARID3BSH. There were no statistical differences between treatment groups.

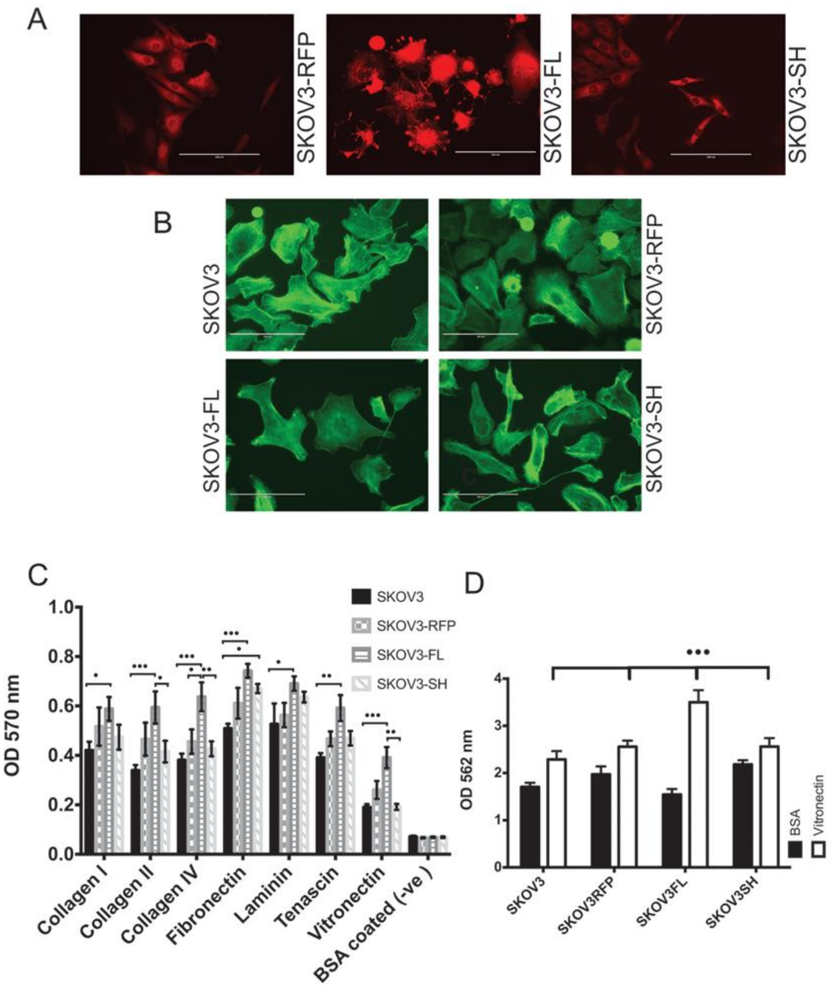

Supplemental Figure 4: ARID3BFL alters cell morphology and increases adhesion, and vitronectin haptotaxis. (A) Live fluorescent microscopy was performed on SKOV3 cells were transduced with lentivirus expressing RFP, ARID3BFL, or ARID3BSH to monitor cell morphology. Original magnification =20x (B) SKOV3, SKOV3-RFP, SKOV3-ARID3BFL, and SKOV3-ARID3BSH cells were stained with Phalloidin to examine the F-actin organization. Original magnification =40x (C) ECM adhesion assays were performed for SKOV3, SKOV3-RFP, SKOV3-ARID3BFL, and SKOV3-ARID3BSH cells. Adhesion to human collagen I, II, or IV, fibronectin, laminin, tenascin, vitronectin, was quantitated. Statistics calculated by two-way ANOVA with repeated measures (* $P \leq 0.05$, ** $P \leq 0.01$, *** $P \leq 0.001$). (D) Vitronectin

haptotaxis assays demonstrate that SKOV3-ARID3BFL cells are significantly more migratory than SKOV3, SKOV3-RFP, and SKOV3-ARID3BSH cells. Migration on vitronectin-coated wells was normalized to the BSA-coated wells. Migratory cells were stained and their absorbance measured and quantitated. Statistics were calculated using a one-way ANOVA and a Tukey's post test (*** $P \leq 0.001$).

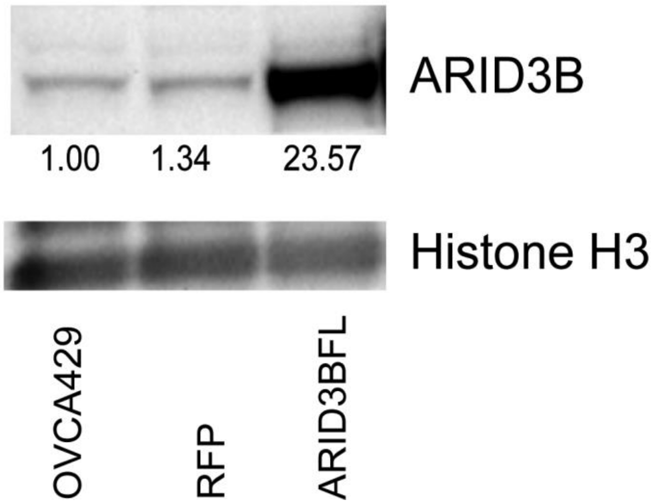

Supplemental Figure 5: Expression of ARID3B in OVCA429 cells. Western blot was conducted for ARID3B on OVCA429 (parental), OVCA429-RFP, and OVCA429-ARID3BFL expressing cells. Histone H3 was used as a loading control. Densitometry was performed in order to assess the level of ARID3B expression compared to the parental OVCA429. Densitometry was normalized to Histone 3 and indicated beneath the ARID3B blot.

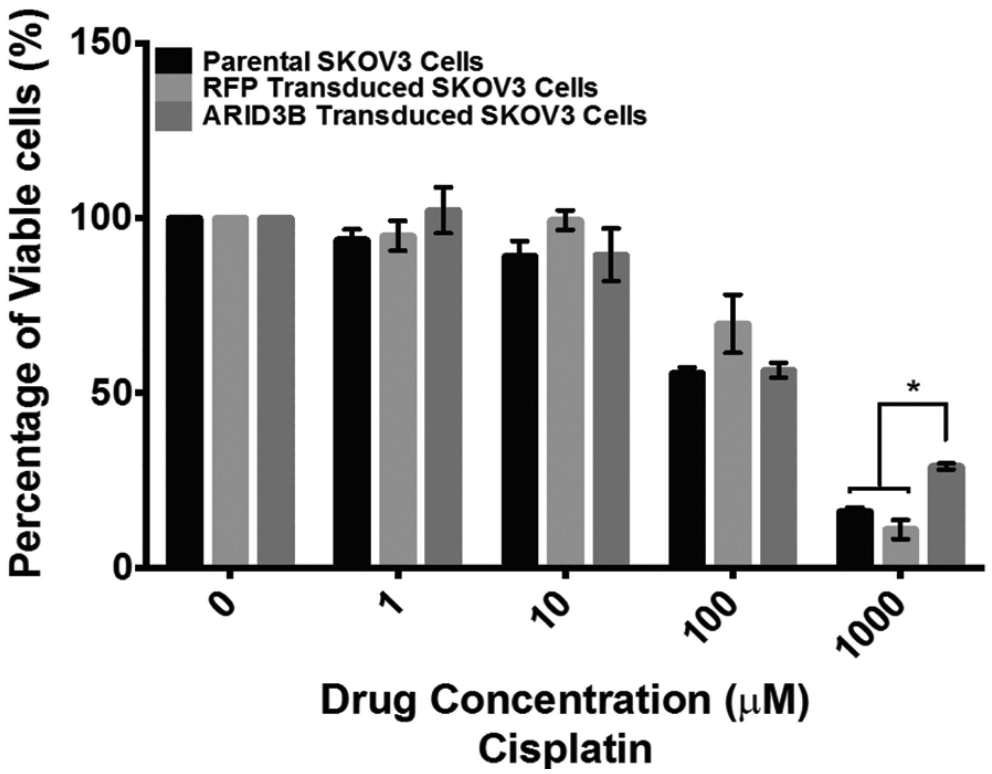

Supplemental Figure 6: ARID3B expression does not affect response to cisplatin. SKOV3, SKOV3-RFP, and SKOV3-ARID3BFL cells were treated with increasing concentrations of cisplatin. MTT assays were performed to measure viability after 48h. The data were analyzed using a Two-way ANOVA. (* $P \leq 0.05$).
